# Cooperative Research and Infectious Disease Surveillance: A 2021 Epilogue

**DOI:** 10.3389/fpubh.2021.817431

**Published:** 2022-01-24

**Authors:** Falgunee K. Parekh, John Hay, Kairat Tabynov, Roger Hewson, Jeanne M. Fair, Sandra Essbauer, Kenneth B. Yeh

**Affiliations:** ^1^EpiPointe LLC, Cary, NC, United States; ^2^Jacobs School of Medicine and Biomedical Sciences, Buffalo, NY, United States; ^3^International Center for Vaccinology, Kazakh National Agrarian Research University (KazNARU), Almaty, Kazakhstan; ^4^Preclinical Research Laboratory With Vivarium, M. Aikimbayev National Scientific Center for Especially Dangerous Infections (NSCEDI), Almaty, Kazakhstan; ^5^Public Health England, Salisbury, United Kingdom; ^6^London School of Hygiene and Tropical Medicine, London, United Kingdom; ^7^Los Alamos National Laboratory, Los Alamos, NM, United States; ^8^Bundeswehr Institute for Microbiology, Munich, Germany; ^9^MRIGlobal, Gaithersburg, MD, United States

**Keywords:** cooperative research, global health security, infectious disease surveillance, scientific publication, COVID-19

## Abstract

As the world looks forward to turning a corner in the face of the COVID-19 pandemic, it becomes increasingly evident that international research cooperation and dialogue is necessary to end this global catastrophe. Last year, we initiated a research topic: “Infectious Disease Surveillance: Cooperative Research in Response to Recent Outbreaks, Including COVID-19,” which aimed at featuring manuscripts focused on the essential link between surveillance and cooperative research for emerging and endemic diseases, and highlighting scientific partnerships in countries under-represented in the scientific literature. Here we recognize the body of work published from our manuscript call that resulted in over 50 published papers. This current analysis describes articles and authors from a variety of funded and unfunded international sources. The work exemplifies successful research and publications which are frequently cooperative, and may serve as a basis to model further global scientific engagements.

## Background

Prior to and during the pandemic, many organizations answered the call to develop and implement assays to detect SARS-CoV-2 in support of improving surveillance for COVID-19. The effective deployment of detection assays relies on robust surveillance and reporting systems. Testing in the absence of a strong surveillance system as a basis will preclude accurate measurement and reporting of the prevalence and incidence of diseases with epidemic and pandemic potential that are necessary to inform public health policy. Global frameworks such as the International Health Regulations, 2005 and the Global Health Security Agenda have enhanced many national and local surveillance systems that incorporate effective diagnostic testing. This work often starts with cooperative and multi-country research, especially those funded by global biosecurity programs, that increase capacity and capability to prevent, detect and respond to epidemics and pandemics (1).

In our 2020 Frontiers research topic: “Infectious Disease Surveillance: Cooperative Research in Response to Recent Outbreaks, Including COVID-19,” we aimed to reinforce the essential synergy between infectious disease surveillance and research. We focused on partnerships, especially involving scientists doing work in countries that are less represented in the scientific literature. Overall, the COVID-19 pandemic has increased awareness, knowledge and know-how for improving infectious disease surveillance and related health systems, as well as cooperative research. Expedited research outputs included detection assays, therapeutics, vaccines, and implementing interventions such as wearing face coverings (2). Recognizing the challenges in quickly operationalizing and implementing research advances to enhance infectious disease surveillance and mitigate the pandemic, our analysis here focuses on the ability to develop and publish scientific literature during an unprecedented year that affected everyone's daily lives. We encouraged the submission of papers that described surveillance and treatment of seasonal and/or endemic diseases (e.g., respiratory, arboviral), and how such work was pivoted to address various aspects of COVID-19. In addition, submission was encouraged from scientists in low-middle income countries and those publishing largely unfunded work from countries less represented in the scientific literature. We anticipate that the lessons learned through the manuscripts published in our topic will improve infectious disease surveillance and cooperative research capacities, such as rapid detection, for emerging infectious diseases and endemic diseases in general.

## Summary of Articles Submitted

The research topic was initiated on April 29, 2020 in Frontiers Journal in the early stages of the pandemic. At that time, we could not have predicted that we would still be combatting the pandemic more than 18 months later. Similar to other research topic calls in Frontiers, which are open for an average of 9–12 months, our research topic remained open for about a year, officially closing submissions on April 5, 2021.

**Figure 1:**
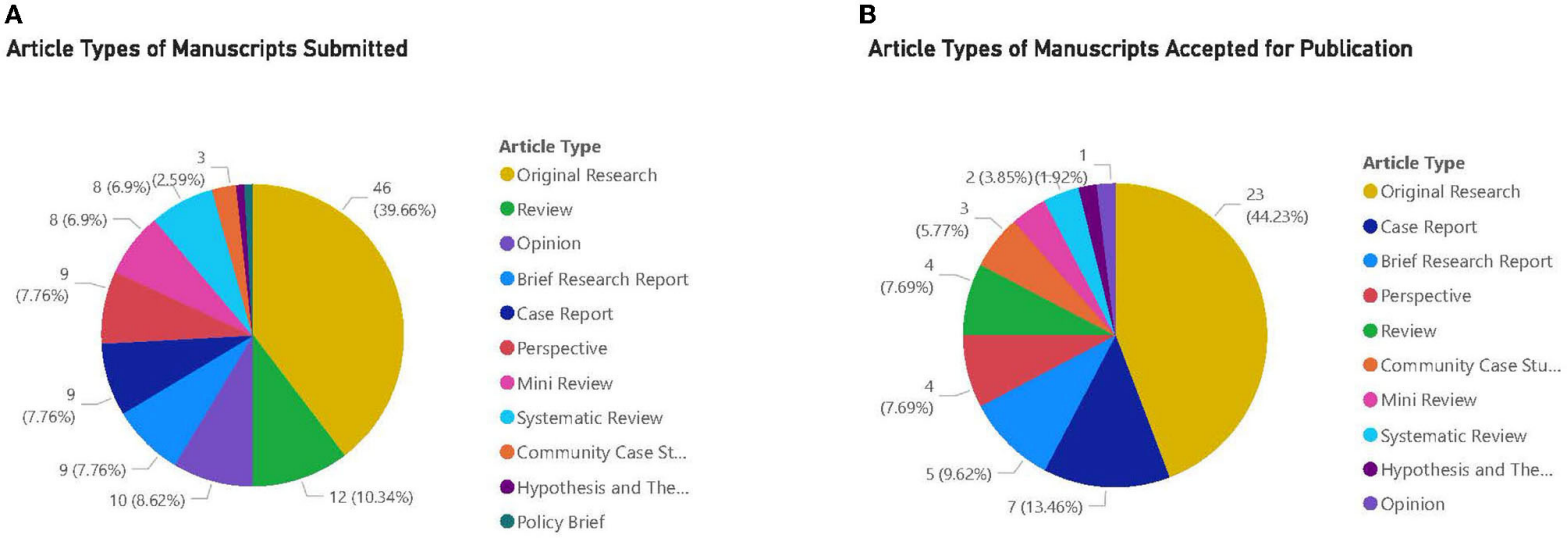
**(A)** Article types of manuscripts submitted. **(B)** Article types of manuscripts accepted for publication. **(A,B)** The majority of articles submitted and accepted for publication were Original Research.

There were 116 manuscripts submitted to the research topic and, to date (Sept 1, 2021), there have been 155,736 total views. The majority of submissions occurred in the first 3 months of the topic call with 84 out of 116 papers submitted May–July 2020. For the remaining months we continued to have 2–5 new submissions each month. There were 11 types of articles that could be submitted, and Figures 1A,B shows the breakdown of total articles submitted and accepted by type of article, respectively.

**Figure 2:**
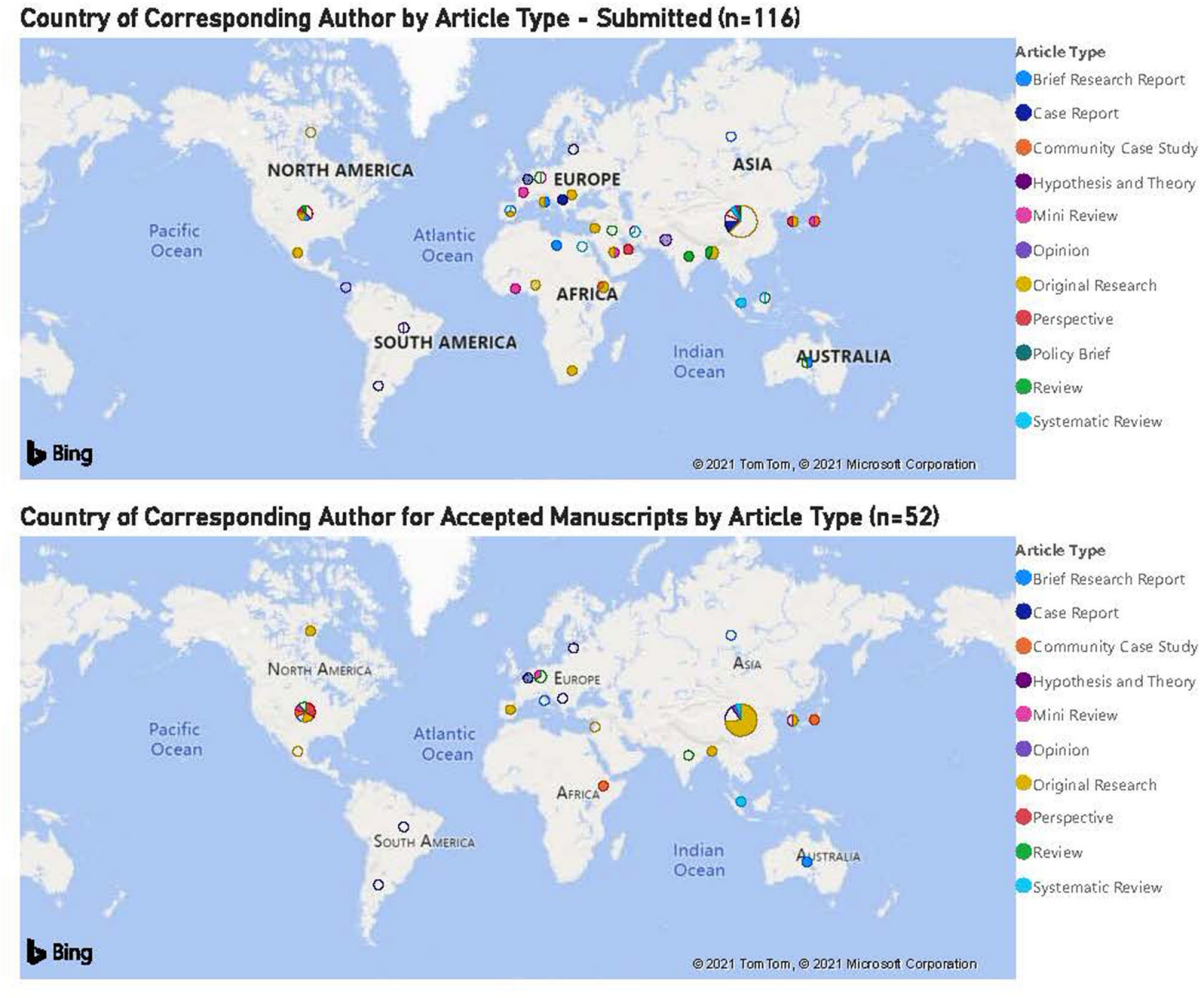
Country of corresponding author for accepted manuscripts by article type (*n* = 52). The top three country contributions were from China, USA, and Germany.

There was substantial and significant global representation in manuscript submissions with corresponding authors from 37 different countries (Figure 2). Numerically, the top three country contributions were from China, The USA and Germany, either from that country alone or in collaboration with another country or countries. Thirty-seven percent (43/116) of article submissions came from corresponding authors in China, of which 27 were Original Research manuscripts. The majority of papers with the corresponding author from China were COVID-related, reflecting, presumably, the level of research interest in COVID in China, especially during the early months of the pandemic.

**Figure 3:**
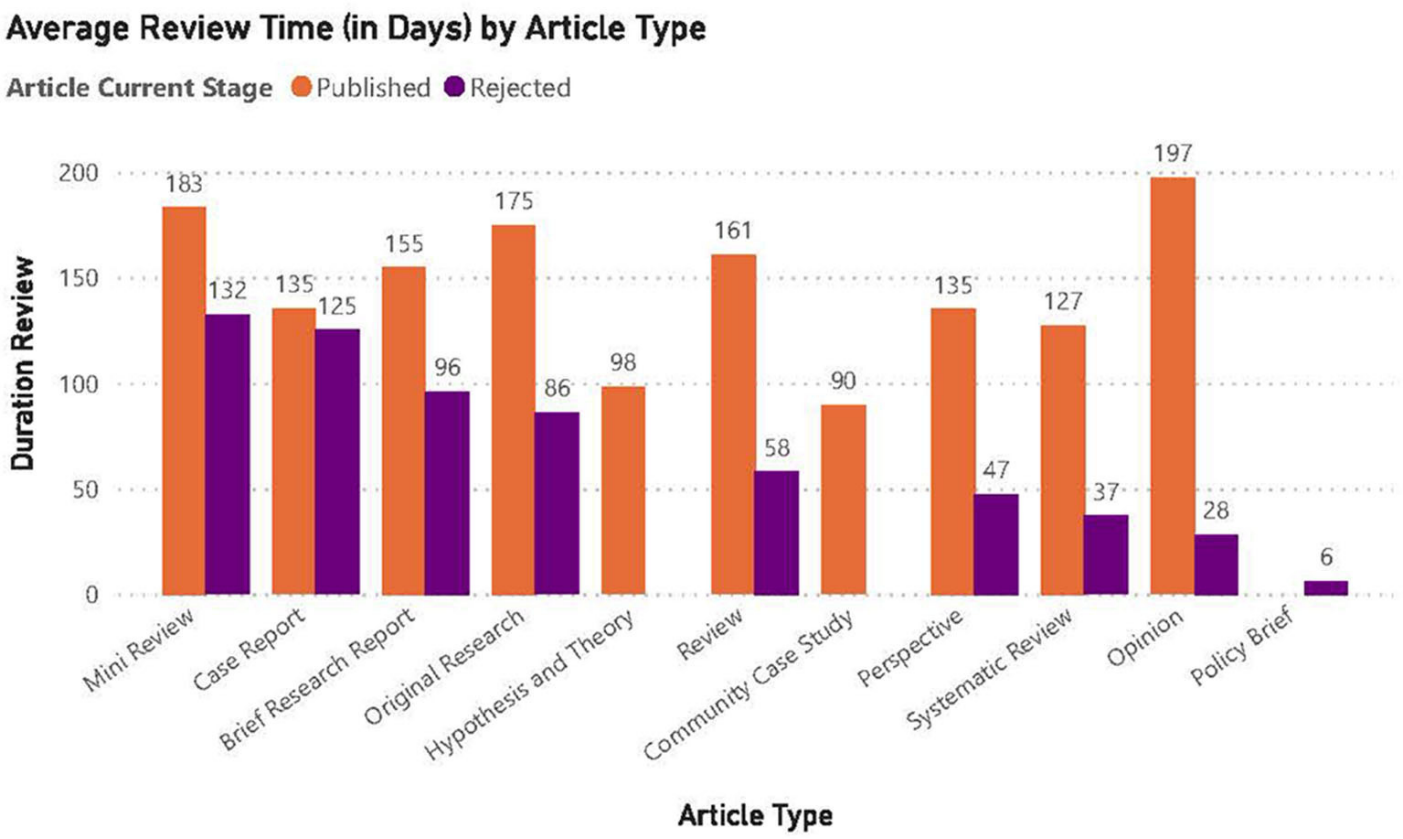
Average review time (in days) by article type. The average review time for manuscripts accepted for publication was 157 days.

Of the 116 manuscripts submitted 52 were accepted for publication. The average review time for published articles was longer at about 157 days compared to those not accepted for publication at about 72 days. The trend of longer review times for published articles vs. not accepted articles was consistent across all article types (Figure 3). The shorter time probably reflected the lack of to-and-fro correspondence normally associated with the full review process.

Corresponding authors from China had the greatest number of manuscripts accepted for publication (*n* = 19) and the USA was second (*n* = 9), consistent with the number of manuscript submissions.

Most of the articles (38/52) accepted for publication dealt with SARS-CoV-2/COVID-19. Other subjects of published articles are Rickettsiae, Understanding quarantine, Entrepreneurial activity, Hemorrhagic fever with renal syndrome, Chikungunya, Measles, West Nile virus, Acute respiratory illness, Infectious diarrhea, Cooperative programs, Klebsiella, and Molecular detection.

## Discussion

Our research topic garnered substantial international attention. The large number of submissions is consistent with a recent analysis of publication patterns related to the pandemic, which showed a sharp increase in publication volume since the onset of COVID-19 (3). According to a recent report, researchers emphasize the importance of learning lessons and developing resilience in the COVID-19 pandemic and note that remote working has allowed further opportunities to develop scientific publications (4). In addition to an increase in publication volume related to the pandemic, it was also noted that COVID-related manuscripts had a significantly faster time to acceptance compared to non-COVID papers (3). For our research topic we did not note a faster time to acceptance. Indeed, the average review time for accepted manuscripts was ~157 days, which was longer than we would have preferred, particularly because of the immediate relevance of many of the manuscripts. Manuscripts that were accepted for publication had longer review time than those not accepted likely because of the time it takes for initial review, authors' revision and responses, and second review. Perhaps the most serious issue for contributors and editors alike was the difficulty in finding suitable reviewers. While in some cases, the pandemic offered some researchers a setting more conducive to writing and publishing work, the same researchers are often requested to peer-review other work. Often, manuscripts would languish for months before two reviews were obtained. We attribute this mainly to the fact that this call focused on infectious disease, specifically mentioning COVID-19 and, either potential reviewers were busy with COVID-19 related work, and/or were busy writing papers and grant applications themselves. Perhaps in response to long review times, there is a trend for biological and clinical research manuscripts to be submitted as pre-prints on various online portals which provide a forum for public comment; an approach that seems to be more frequently utilized during the pandemic. While there is certainly some value in this approach, in the context of making new data quickly available to a wide readership, there is also the substantial danger that this non-peer-reviewed work, with a higher likelihood of being flawed, will lead to incorrect outcomes.

The majority of manuscripts submitted and accepted for publication under our research topic demonstrated scientific cooperation emphasizing the level of and value in cooperative research and publication. Almost 40% of the contributions to our call involved international cooperation (at least two countries involved). Examples of international cooperation included: The US and Russia; The US, Cyprus, the UK and Denmark; Belarus, Estonia, The US and Abu Dhabi; Germany and China; The US and Kazakhstan; Ghana, Australia and Germany; Canada and Bangladesh; Canada and China; the UK and China; Australia and the United Arab Emirates; Spain Italy and Switzerland; China and the US; Kyrgyzstan, Kazakhstan, The UK and The US; Germany, The US, The UK and Kazakhstan. International collaboration is critical in overcoming the COVID-19 pandemic and preparing for future epidemics and pandemics. In February 2020, WHO, in collaboration with Global Research Collaboration for Infectious Disease Preparedness and Response (GLOPID-R), published “A Coordinated Global Research Roadmap: 2019 Novel Coronavirus” based on the R&D Blueprint Strategy, which re-emphasized global cooperative research as an important aspect to addressing the COVID-19 pandemic (5). International research collaboration enables an avenue for knowledge discovery and technology sharing that can drive improvements and innovation in research activities (6–8). This knowledge sharing has become especially relevant with COVID-19, as we must identify best practices and lessons learned on a global scale in order to effectively address a pandemic.

In addition to the encouraging levels of international cooperation, there were also four examples of first authorship being in the hands of an individual from a country under-represented in scientific literature, with supporting roles by other authors from a more-developed country such as the US. It is significant to see this, as it is an essential by-product of the scientific/medical community's efforts to cooperate with and train colleagues from those countries. Several manuscripts described cooperation within one country, however. For example, the work described in Brazil involved five different clinical locations within the country.

The importance of international cooperative research in addressing epidemics and pandemics is not a novel concept. Since the emergence of SARS in 2003, there have been consistent increases in international collaborations during various epidemics, including H1N1, Ebola, and Zika. The COVID-19 pandemic has re-invigorated the importance of having a strong infrastructure in infectious disease surveillance, and has re-emphasized that cooperative research, especially those funded by global biosecurity programs, is a critical element in enhancing capacity to effectively prevent, detect and respond to epidemics and pandemics. Scientific publications that highlight the important outputs from international cooperative research should continue to be prioritized. Through this activity, networks and communities will be further galvanized and better prepared for the next infectious disease threat.

## Data Availability Statement

The original contributions presented in the study are included in the article/supplementary material, further inquiries can be directed to the corresponding author/s.

## Author Contributions

KY, FP, and JH developed this concept along with KT, RH, JF, and SE. All authors reviewed and agreed on the final submission.

## Conflict of Interest

FP was employed by the company EpiPointe, LLC. The remaining authors declare that the research was conducted in the absence of any commercial or financial relationships that could be construed as a potential conflict of interest.

## Publisher's Note

All claims expressed in this article are solely those of the authors and do not necessarily represent those of their affiliated organizations, or those of the publisher, the editors and the reviewers. Any product that may be evaluated in this article, or claim that may be made by its manufacturer, is not guaranteed or endorsed by the publisher.
